# Rapamycin co-exposure fails to reduce cisplatin-induced damage in GC6-spg spermatogonial cell line

**DOI:** 10.1530/RAF-26-0044

**Published:** 2026-07-28

**Authors:** Wafiya Jibrin, Rod T Mitchell, Federica Lopes

**Affiliations:** ^1^ School of Medicine, University of Dundee, Dundee, UK; ^2^ Centre for Vaccine Research and Biotechnology, Federal University Teaching Hospital Lafia, Lafia, Nigeria; ^3^ Centre for Reproductive Health, University of Edinburgh, Edinburgh, UK; ^4^ Royal Hospital for Chidren and Young People, Edinburgh, UK

**Keywords:** rapamycin, cisplatin, chemotherapy, infertility

## Abstract

In the UK, every day about five children receive a diagnosis of cancer. Although the majority (over 80%) will survive, some will become infertile depending on the severity of the treatment received during childhood. Currently, there are no medical treatments available to protect male fertility. This study assessed if rapamycin, a drug currently used to treat other medical conditions, which has been shown in experimental studies to help protect female fertility from cancer treatment, could be used to protect male fertility. Results showed that rapamycin did not prevent overall loss and damage of the cells that will develop into sperm. Further studies are needed to find medical strategies to preserve young boys’ fertility.

Fertility preservation of boys undergoing cancer treatment remains an important clinical consideration given the risk of long-term infertility. Spermatogonial stem cells, essential for spermatogenesis during adulthood, are sensitive to gonadotoxic chemotherapy. Current fertility preservation strategies include testicular tissue cryopreservation before treatment for future use to restore fertility; however, approaches to protect spermatogonia from chemotherapy-induced damage remain limited.

Rapamycin, an mTOR inhibitor approved for clinical use, protects female fertility from chemotherapy toxicity in mice (Goldman *et al.* 2017, Xie *et al.* 2020), by preventing mTOR-mediated premature ovarian follicle activation (Zhang *et al.* 2022). Given the central role of mTOR signalling in cell survival and proliferation, it represents a potential target for protecting male germ cells. Here, we performed a preliminary investigation to assess whether rapamycin could protect spermatogonia from damage and loss induced by cisplatin, a chemotherapeutic agent frequently used in paediatric oncology.

GC6-spg cells, derived from rat type-A spermatogonia, were cultured as described (van Pelt *et al.* 2002). On day 0, cells were seeded at a density of 37.5 × 10^3^/cm^2^. On day 1, phase-contrast images were captured using IncuCyte® Zoom prior to 24 h exposure to a clinically relevant cisplatin concentration (1 μg/mL), a range of rapamycin doses (0.01–20 μM; Biosynth), their combination, or vehicle control (three technical replicates). On day 2, media were replaced with drug-free media containing 3 μM of cleaved caspase-3/7 apoptosis biomarker (NucView® 488). On day 3, propidium iodide (PI, 3 μg/mL) was added 30 min before phase-contrast and fluorescent imaging. Data were analysed using two-way ANOVA (mixed-effects model) with post hoc Tukey test, comparing rapamycin doses with vehicle control and combination treatments with cisplatin alone at the same time point (day 3 and day 6) and the same experimental condition between day 3 and day 6. Independent experiments (*n* = 5–8) were analysed for statistical significance (95% CI; *P*-value ≤ 0.05).

Rapamycin did not alter GC6-spg cell confluency compared with control, with only the highest concentration (20 μM) inducing a significant difference between culture days (Fig. 1A). Cisplatin significantly reduced cell confluency on day 6 compared with control, and this was not rescued by rapamycin co-treatment (Fig. 1B). The highest concentration of rapamycin increased apoptosis on day 3; however, this was reduced on day 6 compared with day 3 (Fig. 1C). Cisplatin-induced apoptosis on day 3 and day 6 was not attenuated by rapamycin co-treatment (Fig. 1D). Rapamycin treatment did not change membrane integrity (Fig. 1E). Cisplatin increased membrane damage on day 6, but this was not significant (Fig. 1F). Within the same treatment conditions, membrane damage was significantly higher on day 6 compared with day 3 in most co-treated cells (Fig. 1F).

**Figure 1:**
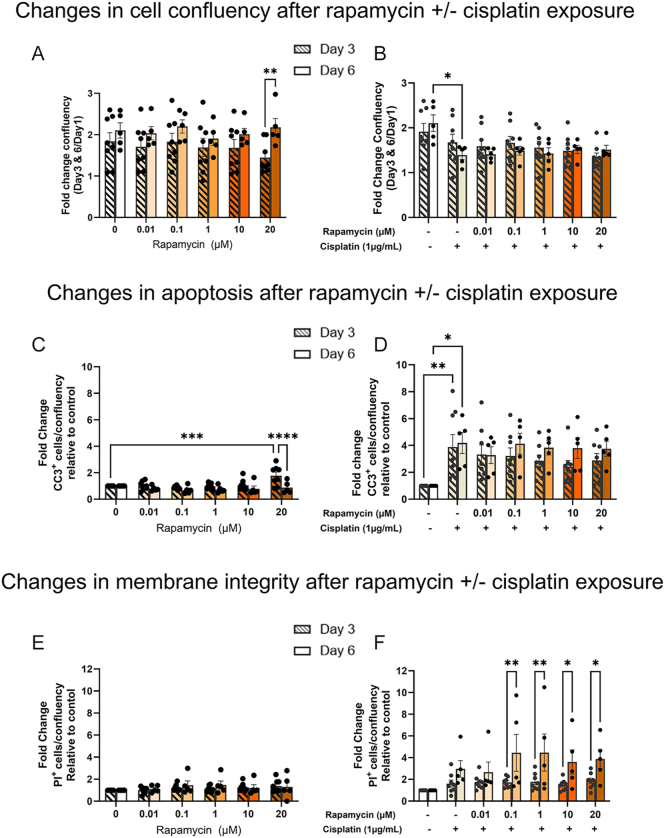
Effect of rapamycin and cisplatin concentrations on a spermatogonial stem cell line (GC6-spg). Cell confluency (A and B), apoptosis – CC3/7^+^ (C and D) and membrane damage – PI^+^ (E and F) were assessed following treatment with increasing concentrations of rapamycin alone (A, C, E) or in combination with cisplatin (B, D, F) at day 3 and day 6 of culture. Data are presented as mean ± SEM (*n* = 5–8 independent replicates, each from the mean of three technical replicates). Statistical analysis was performed using two-way ANOVA with *post hoc* Tukey test, comparing treatment with control group and each experimental group across culture days. *P ≤ 0.05, **P ≤ 0.01, ***P ≤ 0.001, ****P ≤ 0.0001.

Overall, rapamycin did not change GC6-spg confluency, confirming results of an *in vivo* mouse study (Busada *et al.* 2015). Under the conditions tested, rapamycin failed to mitigate cisplatin-induced cytotoxicity, as reflected by reduced confluency, increased apoptosis and loss of membrane integrity, contrasting with rapamycin-mediated protection reported in female models and possibly reflecting different roles for mTOR signalling in the gonads (La *et al.* 2022). These results should be interpreted with caution; experiments were conducted using an immortalised cell line derived from adult rat spermatogonia using a single cisplatin concentration and acute co-treatment regimen. Further studies are warranted to explore fertility preservation options for boys.

## Declaration of interest

F Lopes is an Associate Editor of *Reproduction & Fertility* and was not involved in the peer review or editorial process for this paper, on which she is listed as an author. The authors have no conflict of interest to declare.

## Funding

This study was supported by Medical Research Foundation to FL (MRF-045-0001-RG-LOPE-C0905, code 119608). RTM was supported by a UK Research and Innovation (UKRI) Future Leaders Fellowship (MR/S017151/1). For the purpose of open access, the author has applied a Creative Commons Attribution (CC BY) licence to any Author Accepted Manuscript version arising from this submission.

## Author contribution statement

WJ performed the experiments, analysed the data and contributed to manuscript drafting. RTM helped design the study and interpret the data and revised the manuscript. FL planned and supervised the study, analysed the data and wrote the manuscript. All authors approved the final manuscript.
